# Communal proactive coping strategies among Tamil refugees in Norway: A case study in a naturalistic setting

**DOI:** 10.1186/1752-4458-5-9

**Published:** 2011-04-26

**Authors:** Eugene Guribye, Gro Mjeldheim Sandal, Brit Oppedal

**Affiliations:** 1 Division of Mental Health, Norwegian Institute of Public Health, P.O. Box 4404 Nydalen, 0403 Oslo, Norway; 2 Department of Psychosocial Science, Faculty of Psychology, University of Bergen, P.O. Box 7807, 5020 Bergen, Norway; 3 Regional Centre for Children and Adolescents' Mental Health, P.O. Box 7810, 5020 Bergen, Norway

## Abstract

**Background:**

An exclusive focus on individual or family coping strategies may be inadequate for people whose major point of concern may be collective healing on a more communal level.

**Methods:**

To our knowledge, the current study is the first to make use of ethnographic fieldwork methods to investigate this type of coping as a process in a natural setting over time. Participant observation was employed within a Tamil NGO in Norway between August 2006 and December 2008.

**Results:**

Tamil refugees in Norway co-operated to appraise their shared life situation and accumulate resources communally to improve it in culturally meaningful ways. Long term aspirations were related to both the situation in the homeland and in exile. However, unforeseen social events created considerable challenges and forced them to modify and adapt their coping strategies.

**Conclusions:**

We describe a form of coping previously not described in the scientific literature: C*ommunal proactive coping strategies*, defined as the process by which group members feel collectively responsible for their future well-being and co-operate to promote desired outcomes and prevent undesired changes. The study shows that proactive coping efforts occur in a dynamic social setting which may force people to use their accumulated proactive coping resources in reactive coping efforts. Theoretical and clinical implications are explored.

## Background

The present study focuses on communal proactive coping strategies among Tamil refugees in Norway. Research on coping strategies has tended to focus on how individuals respond to stressors [1-3]. This has entailed two epistemological limitations: 1) *Individuals *may gather social support to cope with their problems, but we have little knowledge about conjoining, interactive group efforts to cope with common problems. 2) Individuals may *respond *to stressors, but we need more knowledge about what people do in *advance *to reduce the likelihood of stressors occurring at all. Acknowledgement of these limitations has resulted in research on communal and proactive coping strategies.

### Communal coping strategies

Collective forms of coping have been conceptualized and studied in a number of distinct scientific disciplines with divergent theoretical and methodological frameworks. Ethnographic studies have focused on collective healing rituals in which individuals are supported and social relations are restored after stressful events [4-6]. Other social scientists have investigated the social resilience of communities rebounding after large scale disasters [7-10]. In organizational studies, there has been a focus on joint coping efforts against work related stress [11,12]. Zhang & Long [13] used Hoefstede's [14] general distinction between individualist and collectivist cultural values as a starting point and defined *collective coping *as activities that orient attention to relationships with in-group members and the maintenance of interpersonal relationships with the goal of managing work-related stress.

A problem with the latter approach is that a great deal of studies reveal that there is more variation regarding collectivist and individualist values within nations than between them [15], suggesting that the dichotomy may be too broadly defined [16]. Furthermore, the epistemological focus on the individual's efforts to establish social supportive relationships within the in-group and conform to in-group norms makes the analytical demarcations between social support [17,18] and collective coping blurry. On a descriptive level, Wong [19] has suggested that in some cases, group-based coping efforts may involve more than providing social support within interpersonal relationships. Members of a group may make a concerted effort to tackle a common problem, for instance the stress of caring for a terminally ill parent. Rather than assisting somebody within their network to cope with a problem, the group cooperates to find a solution to a problem they perceive as *shared*. Asians for instance tend to emphasize collectivistic identity and seek help from social networks rather than from professional counsellors [20-22]. Previous studies show that under distress, Tamil individuals tend to view themselves as integral units of a larger traumatized society, rather than as lonely sufferers [23-25]. These studies show that the family or the community may act to define and interpret the traumatic events, provide mutual support and face the threat as a unit. Collective coping mechanisms may include collectivizing personal trauma, reconstructing meaning and drawing on collective ritual practices that may modify the prevalence of emotional reactions following traumatic events. Rituals may serve to position individuals firmly within social support networks by making grieving a community affair or by focusing on the social obligations that derive from the sacrifices of deceased kin or community members.

Distinctions between social support and collective forms of coping have been further developed in studies on family coping. Families may make use of existing family resources and develop new sets of behaviours and resources to reduce the impact of stressors and strengthen the family unit [26,27]. Lyons, Mickelson, Sullivan, & Coyne [28] use the term *communal coping *to refer to cooperative problem solving processes within the family and other social units dealing with stressful life events. In this model, communal coping and social support are distinguishable along dimensions of appraisal and action. Social support may involve situations in which stressors are appraised as a shared problem, but are mainly perceived as the responsibility of the person who is stressed; or situations in which stressors are appraised as an individual problem, but the responsibility is shared within a social network. Communal coping on the other hand, involves that group members must see the problem as a common problem, must feel collectively responsible for the solution, and must share a commonly desired solution.

Afifi, Hutchinson & Krouse [29] argue that a limitation of the above model of communal coping is that it tends to assume the perspective of the individual support seeker rather than the perspective of the social group. They propose that group norms, rules, and power may dictate the level of ownership and action that group members assume for a stressor. For instance, parents and children often do not have equal legitimate power within a family, and may assume different coping responsibilities. Consequently, we need to employ research methods which enable us to gain better insight on group processes. There has been little focus on the processes and mechanisms by which groups come to appraise problems communally [30]. Another limitation in current research is that studies of communal coping have tended to focus on co-operative problem solving, but there has been less focus on co-operation to realize common goals or attempt to prevent problems from occurring.

### Proactive coping strategies

Research on proactive coping has attempted to broaden our understanding of coping by taking into consideration what occurs in *advance *of stressful [19,31,32]. Whereas individuals may attempt to relieve stress by solving the problem at hand or regulate their emotional responses [33], or by avoiding thinking about the event all together [34] they may also anticipate potential catastrophes before they occur. For instance, they may invest in financial insurance towards natural disasters and store up on supplies, improving their general chances of coping with natural disasters.

Aspinwall & Taylor [31] identify five stages in proactive coping: (1) *Resource accumulation*: Effective proactive coping involves the building of a reserve of temporal, financial and social resources which may make it feasible to manage future stressors. (2) *Recognition of potential stressors*: Potential stressors may be recognized by assessing potential dangers in the environment and remaining sensitive to cues suggesting that threats may arise. (3) *Initial appraisal*: Assessments of current and potential status of these threats. (4) *Preliminary coping efforts*: Activities undertaken to attempt to prevent or minimize the anticipated stressor. (5) *Elicitation and use of feedback concerning initial efforts*: Effects of these efforts are assessed, possibly with the aid of social support network.

However, Schwarzer [32,35] introduces another set of analytical terms, and operates with a distinction between *preventive coping *which aims at preventing potential stressors from occurring (as in the perspective of Aspinwall & Taylor outlined above), and *proactive coping *which in his perspective involves the accumulation of resources in order to realize future goals and ensure quality of life. In comparison with Aspinwall & Taylor's perspective, the focus is not as much on the coping process, but on beliefs that motivate long-term strategic goal-planning. Proactive coping is characterized by positive aspirations and conceptualized as entirely distinct from reactive coping.

Sohl & Moyer [36] investigated whether efforts to build up general resources to ensure quality of life or efforts to prevent or modify potentially stressful events were most predictive of well-being. They assessed proactive coping quantitatively by instruments such as the Proactive Competence Scale (PCS) and the Proactive Coping Inventory (PCI). These self report instruments focus on cognitive processes related to appraising stressful situations, i.e. competencies or skills such as abilities to *Ask for social support*; *Ask for support when things become difficult*; and *Be open for suggestions and advice from others *(PCS) [31,37], and beliefs such as *I visualise my dreams and try to achieve them*; *Before disaster strikes, I am well-prepared for its consequences *(PCI) [32,38]. By using this research design, it was found that only efforts to ensure quality of life (Schwarzer's proactive coping) were distinctly predictive of well-being [36].

However, the role of resources, central to both of the above perspectives on proactive coping, has been inadequately operationalized by Sohl & Moyer. The functions of accumulated temporal, financial and social resources in proactive coping are difficult to assess properly in self-report instruments. We learn that individuals have the competence to ask for social support, but that should not be conflated with knowledge about the resources available to an individual and how these come into play in a proactive coping process. Whether a respondent actually *receives *the social support s/he reports to have the competence to *ask *for, depends upon contextual barriers such as prior input in the relationship [39], and the kind of resources available within the social support network. For instance, if the request for support was motivated by financial challenges, somebody in your support network needs to have either the financial resources to provide a loan; or applicable knowledge on how to solve the situation in other ways. And if this person has vainly asked for your social support previously and kept track of the inputs in the relationship, chances are s/he will be less willing to help you now. The competence to *ask *for social support, then, is merely a small step in a larger process.

The use of standardized checklists to assess respondents' self reports of appraising stressful situations renders an incomplete portrait of coping because they don't grasp the dynamics of the stressful situations [40,41]. Lazarus & Folkman [42] originally viewed coping as an ongoing dynamic process which changes in response to the changing demands of a stressful event. Yet, it has seldom actually been analyzed as such in research [29]. Consequently, there is a need for a broader range of methods to assess coping, and perhaps in particular proactive coping in which people try to prepare themselves for nebulous events that have not yet happened. The myriad of social factors that may give them form is difficult to predict precisely, and planning for events that turn out to be different from those initially envisioned may render proactive coping strategies ineffective [31]. As it is difficult to measure such contextual interplay by psychometric scales a process perspective where changes can be studied over time appears a more appropriate method to examine proactive coping efforts [43].

### Tamils in Norway

Previous studies show that the Tamil community in Norway has been able to downplay traditional class and caste distinctions and organize themselves effectively on the basis of a common nationalist agenda aspiring towards a separate Tamil state in Sri Lanka [44-46]. Consequently, Norwegian Tamils seemed to make a potentially interesting case study of communal proactive coping. The manifestation of Singhalese nationalism in important political acts since Sri Lanka gained independence in 1948 gradually resulted in a full scale civil war between the government and the Tamil separatist organization Liberation Tigers of Tamil Eelam (LTTE) by the 1980's. After a brief cease-fire agreement facilitated by Norwegian diplomats in the early 2000's, hostilities escalated drastically in the last half of the decade, culminating with the military defeat of LTTE in May 2009. Hundreds of thousands of Tamils have left the country during the long-lasting civil war.

Before the war, a small group of Tamils were brought to Norway as labour immigrants, but the majority of Tamils in the country have arrived as refugees. Following Statistics Norway, we employ the term "refugees" to include all persons who came to Norway as a result of flight, regardless of their legal status according to the Geneva Convention. We note however, that among informants, the term "Norwegian Tamils" was preferred. According to Statistics Norway, as of January 2007 there were approximately 12 750 persons with Sri Lankan background in Norway, the majority Tamils. About 6000 live in the capital Oslo, and 1000 live in Bergen, the second largest city. In spite of the traumas they experienced in the past and the adversities they experience in the acculturation process, Tamils in Norway have been considered "model immigrants" who are well integrated in the labour force, have not relied upon social welfare, have experienced little problems related to crime, and their children are among the most successful in the higher education institutions [47,48].

### The current study

The current study aimed at addressing the above outlined limitations in research on coping strategies by conducting a longitudinal investigation in a naturalistic setting of collective efforts to cope proactively. We were interested in investigating how Tamil refugees cope with their life situation *outside *the public services. We expanded on previous definitions [28,37] and conceptualized C*ommunal proactive coping strategies *as the process by which group members feel collectively responsible for their future well-being and co-operate to promote desired outcomes and prevent undesired changes. We followed activities within a Tamil refugee community in Norway to examine: (1) How these Tamils co-operate to form common goals and promote them (2) How these efforts are shaped in relation to a dynamic and ultimately unpredictable social context. To what extent do social events interfere with these coping strategies?

## Methods

### Procedure

In order to situate communal proactive coping strategies within a social context, a qualitative, exploratory approach in which events could be followed in a naturalistic context was considered the most appropriate method. Ethnographic field research may be particularly suited for documenting social life as a process consisting of emergent meanings established in and through social interaction [49]. Accordingly, participant observation was carried out by the first author within the Tamil Resource and Counselling Centre (TRCC) in Oslo and Bergen. Our aim to follow communal proactive coping strategies as they unfold "naturally" over time warranted a longitudinal methodological approach. Consequently, data was collected between August 2006 and December 2008. The dramatic developments in the civil war in Sri Lanka in 2009 remain outside the scope of this study.

The project has been approved by the Norwegian Data Inspectorate and the Regional Committee for Medical and Health Research Ethics (REK). The study did not involve recording of sensitive personal or health information. The names of participants were replaced with pseudonyms in the field notes and have been omitted throughout the article to secure anonymity. The study has also been approved by the TRCC, and was conducted in close cooperation with this organization. Theories, conclusions, and constructs were discussed with key informants throughout the field work. Key informants were given the opportunity to read and comment upon the penultimate version of this manuscript.

### Participants and recruitment

TRCC is one of the most important voluntary Tamil organizations in Norway, providing services to thousands of Tamils through 18 local departments across the country. The centre was first established in Oslo in 1992 by the Tamil Coordinating Committee (TCC), the political branch of LTTE abroad. Hence, most of the participants in this study openly, but to various degrees supported LTTE and the separatist cause. The relationship between activities at the centre and the LTTE are explored in a separate study [25]. Activities at the centre included Tamil language tuition, cultural events, family counselling, courses and seminars, a youth club, a senior club, sports events and homework assistance. The centre also served as an important social arena where Tamils came together, and where members of various Tamil organizations were able to discuss and plan mutual projects, typically during weekends. The staff at TRCC mainly consisted of Tamil parents of both genders who were well-educated, well employed, and around 40 years old. These persons had lived in Norway for 20-25 years, and were identified as resource persons within the Tamil community. Many of them were involved in a number of other Tamil and Norwegian voluntary organizations as well. These resource persons were recruited as key informants in the study, and included 20 individuals: 14 men and 6 women between 25 and 50 years old.

The qualitative study was an extension of and a subproject to a quantitative study to examine risk and resilience among children of immigrants that was being carried out in TRCC during the same period, The Youth, Culture and Competence Study (YCC). The YCC had initiated collaboration with the headmasters of the TRCC to recruit participants and carry out data collection among their members. Recruitment of participants to the qualitative study was embedded in this collaboration. The key informants were motivated to participate in the study by their assessment of the potential benefits of participation for the Tamil community. These benefits included the opportunity to incorporate research staff (first and last author and research assistants) within the Tamil social networks, and to get information about the mental health of Tamil youth. When the mutual interests in collaboration had been established between the research team and the leadership of TRCC, access to the field was made feasible. Further recruitment of informants was characterised by a naturalistic "snowball" effect resulting from following flows of interaction within the organization: key informants recruited informants, who in turn recruited further informants and so forth. This process provided us with information from a broad spectrum of members and users of the centre.

Participants were provided with information about the study and gave oral consent to participate in it. By following flows of interaction between informants, fieldwork of necessity became multi-sited to include a number of social arenas in both cities. Thus, the fieldwork consisted in participating in activities at the resource centre, as well as in sports-arrangements, political seminars, conferences, film-screenings, public demonstrations, rituals, health-camps, cultural events, and various formal and informal meetings involving members of TRCC. The role of the ethnographer in this context ranged from that of a passive observer, to a more active participant who could provide information related to mental health issues, public mental health services, and the Norwegian society in general.

### Method of data collection

Following a naturalistic field approach outlined by Barth [50], and Emerson and colleagues [49], the first author participated in, observed, and made notes of social interaction and events as they occurred in the field. The fieldwork largely followed regular activities at TRCC and consisted of 2-7 hours of participant observation one to three times a week. The informants spoke fluent Norwegian. Additionally, key informants provided translations of discussions that occurred in Tamil. Initial impressions of activities were noted in detail: What the informants talked about; what generated deep concern or engagement; how participants understood, interpreted, and dealt with these issues; and who participated in various activities. This general approach established a broader context of events related to communal proactive coping. Attention was given to how participants organized their time to participate in the collective efforts towards promoting desired outcomes and avoiding undesired changes. Did this entail financial considerations? How did they recruit people to participate in these agendas? In what ways were personal skills and areas of competence shared with others within the community? Furthermore, attention was given to the content of informal discourses, themes of seminars and meetings at the centre, counselling with resource persons outside the centre, and statements issued to the press. How did social events interfere with these coping efforts? What generated interest and concern among the participants? Which group members contributed specifically in appraising potential stressors and deciding upon preliminary coping efforts? The planning and organization of distinct interventions such as courses in family counselling and meetings with representatives of Norwegian health services were followed closely. Importantly, attention was not merely given to the activities the participants engaged in throughout the fieldwork, but also on the particular meanings they attributed to those activities, within their respective contexts.

### Analysis

The analytical process may be described as a continuous pendulum between theoretical (re)assessment and empirical observation [51]. The general approach was partly built on methodological frameworks developed by Corbin & Strauss [52], and further developed by Emerson and colleagues [49]. The stages may be identified as the following:

Immediately following the day's fieldwork, field notes were written up as fully as possible to facilitate sorting, reordering, and coding of the text. Accounts were framed and organized into units in which paragraphs presented coherent moments that structured the description. Extended entries consisted of series of paragraphs organized into discrete units within that day's entry - such as incidents that were noted as particularly important. Additional memos in the text noted theoretical and analytical hunches that were reassessed and developed throughout the fieldwork. Analytic memos that supplemented descriptive field notes were re-read and reconsidered throughout the fieldwork. This informed the focus of observations in the field. Emergent findings were discussed by the authors throughout the fieldwork and assessed in relation to existing theoretical frameworks on coping strategies. Often, these discussions would subsequently result in more meticulous observation of particular events occurring in the field.

After the completion of the fieldwork, field notes were treated as a data set. Themes, patterns, and variations within the record related to the research questions were identified. In-process analyses were elaborated and re-evaluated by subjecting the broad corpus of field notes to close reflection and analysis. This process involved line by line categorization of paragraphs and extended entries. Abbreviated codes were created and written in the margins of the field notes to group the specific events into five categories corresponding with the distinct stages of proactive coping theorized by Aspinwall & Taylor [31] as described above. Non-corresponding events were grouped in a separate category to investigate discrepancies between the theoretical framework and empirical observations. Field notes were then sorted according to these categories, and rearranged into new data sets by collecting together all data fragments that were related to each category. This document was re-read and recoded, developing a series of subcategories in each distinct coping stage. The result was new sets of categories, and new relationships between them that were explored. In this way, a sustained examination of the study questions was provided by linking together a variety of discrete observations. These categories were also assessed against the general understanding that derived from routinely participating in activities at the centre.

## Results

The qualitative data were organized chronologically to provide an account of communal proactive coping strategies as implemented by the Tamil sample. This account also illustrates how various social events influenced and interfered with the coping strategies. We used the theoretical framework of Aspinwall & Taylor [31] as foundation to organize the data. However, in examining coping as a process over time we realized a need of important modifications with respect to the dynamic of the stages as a result of unpredictable adverse events that the participants also had to deal with as they occurred. Firstly, the data implied a need to include *Forming common goals *as an initial stage in the proactive coping process. This is in accordance with the perspective on proactive coping developed by Schwarzer [32,35]. However, while reaching towards goals, group members also needed to tap into their resources to prevent unforeseen potential stressors along the way, in accordance with the perspective on proactive coping developed by Aspinwall & Taylor [31].

Secondly, observing communal proactive coping over time also showed that the stages didn't necessarily follow each other in the order suggested by Aspinwell & Taylor [31]. We therefore present the stages of the coping process according to the order we observed them over time: (1) *Forming common goals*, (2) *Resource accumulation*, (3) *Recognition of potential stressors*, (4) *Initial appraisal*, (5) *Preliminary coping efforts*, and (6) *Recognition of another potential stressor*

### Forming common goals

From the perspective of the Tamil parents at the centre, the primary goals related to their future well-being were twofold. On the one hand, they found it important for themselves and their children to become integrated in the host society. On the other hand, it was a major concern to encourage their children to maintain Tamil identity and become socially engaged in the Tamil struggle in Sri Lanka. These two goals were clearly seen as the common responsibility of the Tamil community:

Everybody felt that they had the responsibility for themselves and future generations to become integrated and at the same time maintain their identity [...]. With this in mind, TRCC was established [53; our translation from Norwegian]

These goals were influenced by both pre-migratory and post resettlement processes. Post resettlement challenges were related to acculturation, overcoming general prejudice, gaining access to the labour market, and maintaining social support systems in the country of resettlement. Importantly, however, the goal of well-being in exile was also closely related to the collective aspirations for improved social conditions for Tamils in the home country. The long-lasting civil war in Sri Lanka posed a continuous concern for most Tamil parents within the TRCC. Their common goal in supporting LTTE, was the establishment of a separate Tamil state, *Tamil Eelam*, in Sri Lanka. It was important for them to encourage their children to become socially engaged in the conflict as civilians. Thus, intelligence, skills, applicable knowledge and social network connections were important both for the integration of Tamil youth in the host society, and for social engagement in the home country.

Group members' experiences from the civil war came heavily into play here. Political acts which partly blocked Tamil access to education and the labour marked in Sri Lanka were frequently discussed at the centre. Informants stressed that the "Sinhala only" policy of 1956 had made Sinhala the only official language in Sri Lanka, and effectively closed opportunities in the public service for many Tamils. Later, the "standardisation" of exam scores for admission to the universities in 1970 was said to have barred many Tamil youths from attending university. Tamils at the centre who had studied at universities in Sri Lanka accounted that they had been targeted by Sinhalese mobs during the 1983 "Black July" riots in Colombo in which thousands of Tamils were killed and many more forced to leave their homes:

I attended the University of Colombo, first year. There was some racism and difference in treatment, but I didn't care much. Then came *Black July*. Many of my friends at the University were killed by their fellow students. Our family house was burnt down, but we were warned by a Singhalese neighbour in advance and managed to escape. I recall how people chased our car with guns, I will never forget it. We hid for three days at a factory without food. Eventually we found refuge in a school which served as a provisional refugee camp, and travelled to Jaffna. But after a while I returned to Colombo with my father. What made the strongest impression on me was that our neighbours had piled all my books on top of my desk in our back yard, there was a lot of envy, you see, and these objects were hard to come by in Sri Lanka. And then they burnt everything. At that point, I decided to complete my education and become a professor, no matter what. I returned to the University and explained my situation, but was met with little understanding. I had to complete the entire first year again. That was when I applied for admission at Universities in 25 countries, and eventually I was admitted in Norway. (Male informant in Bergen, translated from Norwegian)

According to informants, many Tamil schools in Sri Lanka were used as refugee camps for internally displaced families at the time of the fieldwork. The blockade of the main traffic artery into the Jaffna peninsula since 2003 had made supplies for the remaining schools in the Northern areas scarce [54]. Accordingly, Tamil parents in Norway were aware that access to education was a scarce good in their homeland:

In Sri Lanka it was only through education one could obtain respect in the society. Here in Norway, you get respect no matter what education you have. But many tend to think long term, that their children will return to Sri Lanka one day, and that is why they want their children to have an education. And here in Norway we have the possibility to get an education, so why not grab it? I think it is positive. In Sri Lanka now, it is like "how can I ask my mother for money for books when we don't have money for food?" (Male informant in Bergen, translated from Norwegian).

Thus, the group members' experiences with hardship in the home country formed part of a common history which to a large extent seemed to influence their desired future life trajectory. This is true both for maintenance of own identity and civil engagement in the Tamil cause in Sri Lanka, and for integration into the host society by participating in education and work force. These goals were partly formed by the LTTE and their political branch abroad, the Tamil Coordinating Committee (TCC) which was instrumental in establishing the centre. However, from the perspective of members at the centre they were all part of the LTTE in the sense that they shared the responsibility for the future well-being of the Tamil people and shared a commonly desired solution.

"I don't *do *anything for them [the LTTE], but if you think about justice and children starving, and the bombs, and if you think that is wrong, then you are also LTTE, right? So the majority here at the centre will say that they are the LTTE or feel that they are working for them (Female informant, Bergen, translated from Norwegian).

This solution required that the LTTE cadres remaining in the home country fought on the battlefield while members of the exile community provided intelligence and financial resources to help develop LTTE-controlled areas and realize their common goal of a separate Tamil state.

### Resource accumulation

Many members invested a lot of time and effort into voluntary work at the centre for the benefit of the Tamil community. By offering tuition, courses, seminars and organizing meetings, knowledge and skills were distributed among the members. In particular, resources were marshalled to improve the performance of Tamil children within the public educational system. The children's achievements in school and in higher education were closely monitored by their parents. If students for instance complained about difficult physics courses, senior students or parents with a University degree in physics would offer additional tuition during the weekends. Each year, motivational seminars were arranged to inform students about trends in the labour market and pros and cons of particular educational institutions. Qualified young Tamils would also be encouraged to make use of the social networks of these resource persons to eventually secure an entry into the labour market. The general idea was that by virtue of higher education, Tamil youth could get the benefits of a good job and come into possession of resources which could be made collectively available. Many Tamil parents tended to encourage their children to become doctors or engineers, two traditionally respected professions in Sri Lanka, but also central in Diaspora development and health projects in Sri Lanka. Figures from the Norwegian Tamil Health Organization (NTHO) suggest that around 1500 of the Norwegian Tamils are educated and employed within the health sector - more than 10 percent of the entire Tamil population in the country. Each year, Tamil "Health camps" were arranged by some of these health care workers from all over the country. During these Health camps, Tamil specialists, general practitioners, nurses, dentists, and at one point a psychologist offered their assistance for the benefit of Tamil patients. Tamil patients came from all over the country to take advantage of their welcomed services. Some patients arrived from other parts of Scandinavia and even from continental Europe, preferring to be treated by Tamil health personnel rather than local practitioners.

Unlike in public health services, the staff put aside as much time as necessary for individual consultations, striving to understand the life situation of the family members. Emphasizing a cultural understanding of the patient's physical and mental health, Tamil doctors would translate the specific problems of the Tamil patients into a language which would be understandable within Norwegian public health services. This would then be formulated into a report which could help the families with further navigation within the public health- and welfare system. In these ways the centre proactively provided social resources that were collectively available to the Tamil community. Sacrificing their leisure time for voluntary communal work, key resourceful Tamils would thus redistribute their individual social resources within the Tamil community. During this process they became role models for the next generation of Tamils who were hoped to eventually follow their example.

### Recognition of potential stressors

In 2007, two events caused a major disruption in the activities at the centre. In April, a research report suggested that many Tamil girls in Oslo were burdened with suicide attempts and mental health problems [44]. The report was described as "a bomb" within the Tamil community, causing enormous stir even if many of them questioned the reliability of the findings. Then, in August, Norwegian media reported a blood-stained showdown between Tamil youth-gangs in Oslo involving the use of Samurai-swords. A series of reports followed in the Norwegian and Sri Lankan media during the following weeks, indirectly connecting the Tamil community to gang-related violence.

### Initial appraisal

Combined, these two events created tangible links between individual and collective well-being among the Norwegian Tamils. The research report was discussed throughout the Tamil community. In internet forums Tamil youth discussed whether there was too much pressure within the Tamil community to become doctors. One of the authors of this article was invited to a call-in program by the Tamil radio, to respond to questions and inform about mental health related issues in adolescents. The resource persons at the centre held several meetings to discuss the report. They particularly talked about the suggestion that the mental health problems among young Tamil girls could be connected to too much social control among Tamil parents or too much emphasis on education. Another key issue was whether the findings were valid and reliable. The researchers of the report were invited to the annual TRCC conference to discuss the findings and possible interventions. The Conference addressed several questions such as: Could the problems discussed in the report reflect natural processes in adolescence combined with worries about the situation in Sri Lanka? Could the point of departure of the researchers have influenced the results? Were the results valid for the rest of the country?

After the Samurai-sword incident, these two events were appraised in relation to each other. Whereas the violent incident was partly viewed as the result of the influx of Tamil youth gangs elsewhere in Europe, there was general agreement that problems among Tamil adolescents in Norway represented a potential problem for the future. Events among Norwegian Tamils had been followed with a particular interest in Sri Lanka for some time as a result of the Norwegian facilitator role in the cease fire agreement. Many Singhalese had questioned the objectivity of the Norwegian facilitators [55]. For instance, suggesting that Norwegian facilitators had been partial to the LTTE, a former president of Sri Lanka once characterized Norway as a "nation of salmon-eating busy-bodies" [56]. Furthermore, the Norwegian flag had been publically burnt in demonstrations outside the Norwegian embassy in Colombo, and hundreds of Buddhist monks had demonstrated against the Norwegian presence in the country [57].

Asian media, including the news site of the Sri Lankan Ministry of Defence, reconceptualised the gang-related violence in Oslo as a terrorist threat and questioned whether Norwegian facilitators were fit to bring peace to Sri Lanka when they were not even able of keeping peace among Tamils in Norway: "For Erik Solheim [Norwegian Special Envoy in Sri Lanka and Norwegian Minister of International Development at the time] the shooting episode [sic] and the LTTE crime spree in Norway has become a liability" [58]. Notice how the sword-fight had turned into a "shooting episode" and a LTTE "crime spree". Consequently, Norwegian Tamils found themselves caught in a potent symbolical arena in which local actions among adolescents were likely to be reinterpreted outside their context and have a potentially negative influence on the political process in Sri Lanka.

At the same time, Tamils now risked the transformation from "super-immigrants" [59] to "terrorists" in the Norwegian public opinion. Tamils feared that this could have serious consequences, especially in the labour market where Tamils had enjoyed a good reputation and often had been able to secure good job positions. Of course, as already described, it was largely through these positions that Tamil resource persons had been able to provide collectively available resources. Hence, from the perspective of Tamils at the TRCC, these events threatened both individual and collective well-being among both Tamils in exile and in the home country.

### Preliminary coping efforts

Guided by their leaders, the Tamils approached the situation employing communal coping strategies. Assessing the events, two interrelated interventions were considered necessary:. Firstly, a Norwegian Tamil Health Network was established in cooperation between Tamil organizations and the YCC research team of the Norwegian Institute of Public Health. The purpose of the network was to organize a study among Tamil children and adolescents in a number of municipalities to investigate the validity of the mentioned research report on mental health problems among Tamil adolescents in Norway. The intention was to use findings from the new study to plan and implement local interventions. This demanded collaborative effort in order to persuade Tamil children, adolescents and their parents to participate in the study. Several Norwegian-Tamil youth were employed in the study as research assistants to inform and recruit participants and collect quantitative data across the country. Secondly, as a proactive measure, the TRCC leadership decided that some sort of intervention directed towards Tamil families and adolescents was necessary. Lacking the proper competence themselves, Norwegian Tamils wanted to bring in somebody with a combined knowledge on mental health issues and Tamil culture. Finding the right person involved a lengthy search through their social networks:

We inquired with several resource persons within the Norwegian Tamil community, and received a tip about a Tamil in Canada. Allegedly, this person had several years of experience with educating family- and youth counsellors. Personally, he has been a family- and youth counsellor for more than 30 years, and has experience from several countries. We approached him and he proved more than willing to help us with the hidden issues we are facing (Male informant in Oslo, translated from Norwegian).

Since he had experienced and survived traumatic events in Sri Lanka himself, and since he intimately understood cultural and social contexts relevant to exiled Tamils, this person became enormously popular within the community.

For several of us, this was the first time we told about our experience from the horrible war in our home country. It became evident that several of us who participated at the course have experienced many traumas, and that some still live with post-traumatic afflictions (Male informant in Oslo, translated from Norwegian).

However, the strategy did not merely involve finding a teacher, but to make this knowledge collectively available by teaching new teachers. The strategy was to empower the Norwegian Tamil community to handle these issues on their own, collectively.

We have seen women, we have seen some young people individually, I have visited families, people have called me for individual counselling, but most important is the training and that is taking plenty of time. It is not my self teaching them, it is rather giving them the empowerment that is within them that they all can become skilled helpers to the people who are in trouble. (Transcription from interview in Radio Migrapolis, NRK, November 25, 2007).

TRCC and the visiting family counsellor offered a series of courses in family- and youth counselling across the country, and certified a number of new Tamil counsellors that were given the role of resource persons. These counsellors in turn organized a Family and Youth-Counselling group offering their services at the centre. During the distribution of certificates to participants at the courses, a Norwegian local politician informed the participants that the centre had received financial support from the Oslo City Council. She emphasized that this was a symbol of recognition of the importance of the centre. This token of social support within the host society was perhaps particularly important in the context of the Samurai sword fight that had threatened the Tamil's image as well-adapted refugees in the country. At the same time, the resource persons at the centre had worked actively to ensure the Norwegian media that they were cooperating with the police in the investigation of the Samurai-sword incident, and that they were working preventively to avoid further incidents.

Local police have had meetings with several resource persons within the Tamil community. The Tamils want to put an end to the crime before it develops. -Everybody who knows something helps out, says [the] leader at the Tamil Resource and Counselling Centre [60].

### Recognition of another potential stressor

While the Tamils were occupied with these preliminary communal coping efforts, the civil war in Sri Lanka escalated drastically. This crisis in the home country interfered dramatically with the ongoing coping processes. To the surprise of most of these Tamils, the governmental military campaign against the LTTE had been very effective. By December 2008, the remaining forces of LTTE, along with 300 000 Tamil civilians, were trapped on the beaches between Nanthi Kadal lagoon and the Bay of Bengal. No reporters or international aid workers were allowed into the area. The faith of friends and relatives within the area was uncertain, and reliable information was largely substituted with rumours and propaganda. Powerless to invoke action from the international community, the Tamil exile community now devoted all of their attention and collective resources to this potentially devastating stressor. Many were concerned that 30 years of continuous, meaningful struggle would now amount to nothing. This was not a time to consider elicitation of previous coping efforts directed towards comparatively minor challenges in the resettlement country. From the perspective of these Tamils, what was at stake now was Tamil genocide.

## Discussion

This study made use of qualitative ethnographic fieldwork methods and investigated communal proactive coping strategies as they unfolded in a naturalistic setting. Members of a Tamil NGO in Norway defined common goals for their future well-being, which they felt collectively responsible for promoting by accumulating resources collectively. However, when potential stressors were recognized, they also appraised the current situation collectively, and tapped into their collective resources to prevent the situation from escalating. Thus, the analytical distinction made between proactive coping as efforts to ensure quality of life [32], and proactive coping as efforts to prevent undesired changes [31] needs to be reassessed, at least on a group level. Whereas people may set out to accumulate resources to realize their positive aspirations, social events may occur along the way that forces them to *respond *by tapping into these resources to attempt to cope with adversity, disaster or physical impairment that occurs before reaching the goal [41,61]. Individuals and collectives are never fully able to predict the turn of events in their lives, and have to make adjustments as life provides them with unforeseen challenges and opportunities. One of the advantages of communal proactive coping strategies may be that people are able to mobilize resources quickly when potential stressors are detected and assessed collectively.

Researcher's attempts to reduce the complexity of coping assessments may occur at the price of neglecting the uniqueness of situation-specific coping responses [62]. In our study, the onset of certain events made it difficult for people to assess their own preliminary coping efforts and following through with a coping process that had been initiated to deal with another stressful situation. Rather, their primary need was to try to readjust to the new serious events that had taken place. Hence, whereas the goal-promotion/stress-prevention distinction may be important in some contexts [36], it needs to be validated against observational data from studies of how proactive coping strategies unfold in naturalistic settings. Mapping individual dispositions or statements about coping competencies does not provide insight into the complex interplay between people's coping strategies and the influence of social events on these strategies. The complex and often longitudinal temporal pattern of proactive coping thus provides a methodological challenge. As Aspinwall & Taylor [31] note, coping inventories are typically not sensitive to these temporal patterns of coping efforts because they require participants to report coping strategies without regard to the order. The present study made use of ethnographic fieldwork to examine coping processes during a 16 months' time span. Even if the fieldwork for the present study was finished, the Tamil community had not concluded their ongoing coping efforts.

Scholars have argued that an exclusive focus on individual or family coping strategies may be inadequate for people whose major point of concern may be collective healing on a more communal level [63,24]. This may hold particularly true for refugees, who may suffer from loss of family members, separation from their extended families, or have to adjust to social mechanisms in the resettlement country that challenge traditional family structures [64,65]. Studies show that despite frequently cited claims, the vast majority of refugees resettled in Western countries do not develop serious mental health disorders [66]. This suggests that powerful cultural and social mechanisms protect the majority of refugees from developing mental health problems in exile in spite of the traumas they experienced in the past and the adversities they experience in the acculturation process [67]. Dedication to political causes; adherence to religion; and meaningful involvement in the community have been found to be effective coping mechanisms [46,68-70]. What is of importance here is that people may co-operate to appraise a shared life situation and accumulate resources collectively to improve it in culturally meaningful ways. This kind of attention towards the social and cultural basis of appraisals has tended to be missing from coping research [41].

However, the concept of collectives needs to be further refined analytically and properly operationalized in future studies on communal coping. The present study suggests that although Tamil co-operated for their future well-being, a small but resourceful group was most instrumental in defining goals and identifying solutions. Moreover, these persons also interacted with corresponding Tamil resource persons within the transnational Diaspora networks, and felt connected to the struggle of the LTTE for a separate Tamil state in Sri Lanka. Yet, the "solutions" that were selected did not only include the people responsible for the initiative, but also larger contingents of the Tamil community. Consequently, there is a need to identify collectives within collectives and investigate how roles and responsibilities are distributed within communities and groups.

The present study also revealed that although people within a community may co-operate to realize a commonly perceived goal or to prevent a common threat, they may also attempt to enlist the social support of people outside the community for the same purposes. In this case, the Tamil organization co-operated with institutions within the host society such as the police, the city council, and the Norwegian Institute of Public Health. The question whether this co-operation should be characterized as social support or collective coping poses some interesting analytical issues. In many regards, the well-being of immigrant communities within the host society is also the responsibility of host institutions. They share the aspiration of integrating immigrant children within the host society. There is also the question of in-group definition here, since immigrant children born in resettlement countries are often granted resettlement country citizenship. Hence, on this level, the demarcations between social support (i.e. assisting somebody to cope with a problem) and collective coping (i.e. cooperation within a group to find a solution to a shared problem) are blurry and may need further clarification.

Public service providers may benefit from collaborating with the types of local immigrant NGO's described in this article in a number of ways. These NGO's often have the infrastructure to enable health- and social services to reach immigrants within social arenas that are trusted by them. For instance, mental health services may collaborate with immigrant NGO's to map potential needs, avoid cultural pitfalls and reach individuals in need of help within a trusted social arena. In this way, social stigmas that are often associated with mental health services within immigrant communities [71] may be reduced. The NGO's may also assist service providers in facilitating information about distinct public services, and provide specific health-related information that may enable immigrants to prevent future health problems from occurring. Social services may benefit from collaborating with immigrant NGO's in designing educational and labour-market directed programmes for immigrant clients, empowering them to avoid long-term clientification. However, public services should also establish routines for assisting the NGO's in coping with unforeseen social events, disasters and adversity which may drain communal resources that were accumulated for other purposes.

A limitation of the study is that we have not investigated the interplay between individual and communal coping strategies. What are the payoffs of collective efforts vis-à-vis individual efforts? What is the interplay between individual and collective belief systems and competencies? When do communal goals become individual goals? Which individual goals and problems never become communal? Another limitation is that the findings may not be transferable for Tamils outside these organizations who may not have similar access to collective resources and do not necessarily share the same mutual aspirations (i.e. a separate Tamil nation). However, support of Tamil Eelam is widespread among Tamils in Norway. A referendum conducted among Tamils in Norway in 2009 showed that 99 percent of the voters endorsed the call for a sovereign Tamil state in Sri Lanka. Eighty percent of the eligible voters turned out for the referendum [72]. A final limitation is that to some degree, the study employs a Western perspective on coping. The role of culture-specific rituals, beliefs, family dynamics and so forth remain outside the scope of the study. As Fuglerud [45] notes, among exiled Tamils religion is largely irrelevant for organisation outside the immediate family, although exiles are divided between Hindus and Catholics. A study within local Hindu temples or Catholic communities may have complemented our findings and presented a more holistic view of coping among Tamils in Norway. To the extent that cultural rituals did come into play in the NGO's described in this study, they were largely connected with the LTTE, and are covered in detail in a separate study published elsewhere [25].

Future research should address the epistemological challenges identified in this article and investigate how the notion of communal proactive coping strategies may apply to other contexts.

## Conclusions

We describe a form of coping previously not described in the scientific literature: C*ommunal proactive coping strategies*, defined as the process by which group members feel collectively responsible for their future well-being and co-operate to promote desired outcomes and prevent undesired changes. The study shows that proactive coping efforts occur in a dynamic social setting which may force people to use their accumulated proactive coping resources in reactive coping efforts. The study suggests that our analytical understanding of coping may be too rigid, and needs to be validated against observational data from studies on how coping strategies unfold in naturalistic settings.

## List of abbreviations used

LTTE: Liberation Tigers of Tamil Eelam; NTHO: Norwegian Tamil Health Organization; PCI: Proactive Coping Inventory; PCS: Proactive Competence Scale; TCC: Tamil Coordinating Committee; TRCC: Tamil Resource and Counselling Centre; YCC: The Youth, Culture and Competence Study.

## Competing interests

The authors declare that they have no competing interests.

## Authors' contributions

EG conceived of and designed the study, participated in recruiting informants, carried out the ethnographic fieldwork, analyzed the data, and drafted the manuscript. GMS participated in the analysis of the data and helped draft the manuscript. BO was the project leader of the Youth Culture and Competence Study which this study was a part of, helped recruit informants, participated in the analysis of the data and helped draft the manuscript.
